# Supplementary material for: Warming of +1.5 °C is too high for polar ice sheets
Source: Commun Earth Environ. 2025 May 20;6(1):351. doi: 10.1038/s43247-025-02299-w (PMC12092291; doi:10.1038/s43247-025-02299-w)
Supplement: Supplementary file 2 — Supplementary Information [file 43247_2025_2299_MOESM2_ESM.pdf]

Supplementary Table 1: Source data of palaeo-climate conditions, ice sheet response and global mean sea level during past warm periods shown in Table 1 of the main manuscript.

Climate Parameter	Sources
CO_2 values for MIS 5e and MIS 11	Luthi et al. (2008)
CO_2 value for Pliocene	Pagani et al. (2010); Seki et al. (2010); Martinez-Boti et al., (2015); de la Vega (2020)
Global Mean Temp (MIS 5e)	McKay and Overpeck (2011); Otto-Bliesner et al (2013); Shackelton et al (2020); Turney et al. (2020);
Global Mean Temp (MIS 11)	Masson-Delmotte et al. (2010); Lang and Wolff (2011)
Global Mean Temp (Pliocene)	Haywood et al. (2020)
Arctic Temps (MIS 5e)	CAPE members (2006); NEEM members (2013); Capron et al. (2014)
Arctic Temps (MIS 11)[1]	Melles et al. (2012); Cronin et al. (2013); Cronin et al. (2019)
Arctic Temps (Pliocene)[2]	Dowsett et al. (2013)
Antarctic Temps (MIS 5e)	Capron et al. (2014)
Antarctic Temps (MIS 11)	Jouzel et al. (2007)
GrIS extent (MIS 5e)	Dutton et al. (2015a); Alley et al. (2010); Colville et al. (2011); NEEM community members (2013); Hatfield et al. (2016); Yau et al. (2016)
GrIS extent (MIS 11)	Reyes et al. (2014); Hatfield et al. (2016); Robinson et al. (2017); Christ et al. (2023)
GrIS extent (Pliocene)	Dolan et al. (2015); Koenig et al. (2015); Dumitru et al. (2019)
GrIS volume (SLE)	Morlighem et al. (2017)
AIS extent (MIS 5e)	Dutton et al. (2015a); Dutton et al. (2015b); Rohling et al. (2019); Turney et al. (2020); Bartlett et al. (2023); Lau et al. (2023)
AIS extent (MIS 11)	Blackburn et al. (2020)
AIS extent (Pliocene)	Grant et al. (2019)
GMSL values	See Figure 1 and Supplementary Table 1

[1] Data from only a few sites, far fewer data than for MIS 5e

[2] Data from only five sites, far fewer data than for MIS 5e

Supplementary Table 2: Published estimates of global mean sea level (GMSL) from coastal sedimentary archives during past warm periods shown in Figure 1 of the main manuscript

Citation (Fig. 1)	Source	Location	Low (m)	High (m)	Mid (m)	+ range (m)	- range (m)
**1**	Veeh (1966)	Indo-Pacific	2.0	9.0	5.5	3.5	3.5
**2**	Chappell and Shackleton (1986)	Huon	2.0	10.0	6.0	4.0	4.0
**3**	Chen et al. (1991)	Bahamas			6.0		
**4**	Stirling et al. (1998)	Western Australia			4.0		
**5**	Muhs et al. (2002a) QSR	Hawaii & Bermuda	3.0	6.0	4.5	1.5	1.5
**6**	Muhs et al. (2002b) QR	Bermuda, Bahamas, Hawaii, Australia	4.0	6.0	5.0	1.0	1.0
**7**	Hearty et al. (2007)	Global	6.0	9.0	7.5	1.5	1.5
**8**	Blanchon et al. (2009)	Yucatan			6.0		
**9**	Thompson et al. (2011)	Bahamas			6.0		
**10**	Muhs et al. (2011)	Florida	6.6	8.3	7.5	0.9	0.9
**11**	Kopp et al. (2009)	Global	6.6	9.4	8.0	1.4	1.4
**12**	Dutton et al. (2012)	Global	5.5	9.0	7.3	1.8	1.8
**13**	O'Leary et al. (2013)	W. Australia (Quobba Ridge)			9.0		
**14**	Kopp et al. (2013)	Global	6.4	8.8	7.6	1.2	1.2
**15**	Dutton et al. (2015)	Seychelles	5.9	9.3	7.6	1.7	1.7
**16**	Polyak et al. (2018)	Mallorca	5	16	10.5	5.5	5.5
**17**	Dyer et al. (2021)	Bahamas	1.2	5.3	3.8†	2.5^	1.6^
**18**	Barnett et al. (2023)	northern Europe	3.6	8.7	5.7	3	2.1
**19**	Dumitru et al. (2023)	Bahamas	0.4	2.7	1.1†	0.7	1.6
**20**	Raymo & Mitrovica (2012)	Bermuda, Bahamas	6	13	9.5	3.5	3.5
**21**	Roberts et al. (2012)	South Africa			13	2	2
**22**	Chen et al. (2014)	South Africa	8	11.5	9.75	1.75	1.75
**23**	Dowsett & Cronin (1990)	eastern USA (Orangeburg Scarp)			35	18	18
**24**	Wardlaw & Quinn (1991)	Enewetak Atoll	20	25	22.5	2.5	2.5
**25**	Miller et al. (2012)	Combination*			22	10^	10^
**26**	Rovere et al. (2014)	South Africa (De Hoop plain)	1.9	20.5	11.2	9.3	9.3
**27**	Moucha et al. (2017)	eastern USA (Orangeburg Scarp)			15		
**28**	Grant et al. (2019)	New Zealand		25			

		(Whanganui Basin)					
**29**	Dumitru et al. (2019)	Mallorca			20.6	2.8	2.8
**30**	Hearty et al. (2020)	South Africa (De Hoop plain)			13.3	6.4	6.1
**31**	Richards et al. (2023)	Australia			16	5.5	5.6

* Estimate derived from back-stripping in Virginia (USA), New Zealand, and Enewetak Atoll (N. Pacific Ocean), benthic foraminiferal $\delta^{18}O$ values, and Mg/Ca-$\delta^{18}O$ estimates.

† mode of peak GMSL from 10,000 simulations

^ 95% confidence interval

Supplementary References:

Alley, R.B., Andrews, J.T., Brigham-Grette, J., Clarke, G.K.C., Cuffey, K.M., Fitzpatrick, J.J., Funder, S., Marshall, S.J., Miller, G.H., Mitrovica, J.X., Muhs, D.R., Otto-Bliesner, B.L., Polyak, L., White, J.W.C., 2010. History of the Greenland Ice Sheet: paleoclimatic insights. Quaternary Science Reviews 29, 1728-1756.

Austermann, J., Hoggard, M.J., Latychev, K., Richards, F.D., Mitrovica, J.X., 2021. The effect of lateral variations in Earth structure on Last Interglacial sea level. Geophysical Journal International 227, 1938-1960.

Austermann, J., Mitrovica, J.X., Huybers, P., Rovere, A., 2017. Detection of a dynamic topography signal in last interglacial sea-level records. Science Advances 3, e1700457.

Barnett, R.L., Austermann, J., Dyer, B., Telfer, M.W., Barlow, N.L.M., Boulton, S.J., Carr, A.S., Creel, R.C., 2023. Constraining the contribution of the Antarctic Ice Sheet to Last Interglacial sea level. Science Advances 9, eadf0198.

Blackburn, T., Edwards, G.H., Tulaczyk, S., Scudder, M., Piccione, G., Hallet, B., McLean, N., Zachos, J.C., Cheney, B., Babbe, J.T., 2020. Ice retreat in Wilkes Basin of East Antarctica during a warm interglacial. Nature 583, 554-559.

Blanchon, P., Eisenhauer, A., Fietzke, J., Liebetrau, V., 2009. Rapid sea-level rise and reef back-stepping at the close of the last interglacial highstand. Nature 458, 881-885.

CAPE Last Interglacial Project Members, 2006. Last Interglacial Arctic warmth confirms polar amplification of climate change. Quaternary Science Reviews 25, 1383-1400.

Chandler, D., Langebroek, P., 2021. Southern Ocean sea surface temperature synthesis: Part 2. Penultimate glacial and last interglacial. Quaternary Science Reviews 271.

Chappell, J., Shackleton, N.J., 1986. Oxygen isotopes and sea level. Nature.

Chen, F., Friedman, S., Gertler, C.G., Looney, J., O'Connell, N., Sierks, K., Mitrovica, J.X., 2014. Refining Estimates of Polar Ice Volumes During the MIS11 Interglacial Using Sea Level Records from South Africa. Journal of Climate, 141006071055006.

Chen, J., Curran, H., White, B., Wasserburg, G., 1991. Precise chronology of the last interglacial period: 234U-230Th data from fossil coral reefs in the Bahamas. Bulletin of the Geological Society of America 103, 82.

Christ, A.J., Rittenour, T.M., Bierman, P.R., Keisling, B.A., Knutz, P.C., Thomsen, T.B., Keulen, N., Fosdick, J.C., Hemming, S.R., Tison, J.L., Blard, P.-H., Steffensen, J.P., Caffee, M.W., Corbett, L.B., Dahl-Jensen, D., Dethier, D.P., Hidy, A.J., Perdrial, N., Peteet, D.M., Steig, E.J., Thomas, E.K., 2023. Deglaciation of northwestern Greenland during Marine Isotope Stage 11. Science 381, 330-335.

Colville, E.J., Carlson, A.E., Beard, B.L., Hatfield, R.G., Stoner, J.S., Reyes, A.V., Ullman, D.J., 2011. Sr-Nd-Pb isotope evidence for ice-sheet presence on southern Greenland during the Last Interglacial. Science 333, 620-623.

Cronin, T.M., Keller, K.J., Farmer, J.R., Schaller, M.F., O'Regan, M., Poirier, R., Coxall, H., Dwyer, G.S., Bauch, H., Kindstedt, I.G., Jakobsson, M., Marzen, R., Santin, E., 2019. Interglacial Paleoclimate in the Arctic. Paleoceanography and Paleoclimatology 34, 1959-1979.

Cronin, T.M., Polyak, L., Reed, D., Kandiano, E.S., Marzen, R.E., Council, E.A., 2013. A 600-ka Arctic sea-ice record from Mendeleev Ridge based on ostracodes. Quaternary Science Reviews 79, 157-167.

de la Vega, E., Chalk, T.B., Wilson, P.A., Bysani, R.P., Foster, G.L., 2020. Atmospheric CO(2) during the Mid-Piacenzian Warm Period and the M2 glaciation. Sci Rep 10, 11002.

Dolan, A.M., Hunter, S.J., Hill, D.J., Haywood, A.M., Koenig, S.J., Otto-Bliesner, B.L., Abe-Ouchi, A., Bragg, F., Chan, W.L., Chandler, M.A., Contoux, C., Jost, A., Kamae, Y., Lohmann, G., Lunt, D.J., Ramstein, G., Rosenbloom, N.A., Sohl, L., Stepanek, C., Ueda, H., Yan, Q., Zhang, Z., 2015. Using results from the PlioMIP ensemble to investigate the Greenland Ice Sheet during the mid-Pliocene Warm Period. Climate of the Past 11, 403-424.

Dowsett, H., Cronin, T.M., 1990. High eustatic sea level during the middle Pliocene: Evidence from the southeastern US Atlantic Coastal Plain. Geology 18, 435-438.

Dowsett, H.J., Robinson, M.M., Stoll, D.K., Foley, K.M., Johnson, A.L.A., Williams, M., Riesselman, C.R., 2013. The PRISM (Pliocene palaeoclimate) reconstruction: time for a paradigm shift. Philosophical Transactions of the Royal Society A: Mathematical, Physical and Engineering Sciences 371, 20120524-20120524.

Dumitru, O.A., Austermann, J., Polyak, V.J., Fornos, J.J., Asmerom, Y., Gines, J., Gines, A., Onac, B.P., 2019. Constraints on global mean sea level during Pliocene warmth. Nature 574, 233-236.

Dumitru, O.A., Dyer, B., Austermann, J., Sandstrom, M.R., Goldstein, S.L., D'Andrea, W.J., Cashman, M., Creel, R., Bolge, L., Raymo, M.E., 2023. Last interglacial global mean sea level from high-precision U-series ages of Bahamian fossil coral reefs. Quaternary Science Reviews 318.

Dutton, A., Lambeck, K., 2012. Ice volume and sea level during the Last Interglacial. Science 337, 216-219.

Dutton, A., Carlson, A.E., Long, A.J., Milne, G.A., Clark, P.U., DeConto, R., Horton, B.P., Rahmstorf, S., Raymo, M.E., 2015a. Sea-level rise due to polar ice-sheet mass loss during past warm periods. Science 349, aaa4019.

Dutton, A., Webster, J.M., Zwartz, D., Lambeck, K., Wohlfarth, B., 2015b. Tropical tales of polar ice: evidence of Last Interglacial polar ice sheet retreat recorded by fossil reefs of the granitic Seychelles islands. Quaternary Science Reviews 107, 182-196.

Dyer, B., Austermann, J., D'Andrea, W.J., Creel, R.C., Sandstrom, M.R., Cashman, M., Rovere, A., Raymo, M.E., 2021. Sea-level trends across The Bahamas constrain peak last interglacial ice melt. Proc Natl Acad Sci U S A 118.

Grant, G.R., Naish, T.R., Dunbar, G.B., Stocchi, P., Kominz, M.A., Kamp, P.J.J., Tapia, C.A., McKay, R.M., Levy, R.H., Patterson, M.O., 2019. The amplitude and origin of sea-level variability during the Pliocene epoch. Nature 574, 237-241.

Hatfield, R.G., Reyes, A.V., Stoner, J.S., Carlson, A.E., Beard, B.L., Winsor, K., Welke, B., 2016. Interglacial responses of the southern Greenland ice sheet over the last 430,000 years determined using particle-size specific magnetic and isotopic tracers. Earth and Planetary Science Letters 454, 225-236.

Haywood, A.M., Tindall, J.C., Dowsett, H.J., Dolan, A.M., Foley, K.M., Hunter, S.J., Hill, D.J., Chan, W.-L., Abe-Ouchi, A., Stepanek, C., Lohmann, G., Chandan, D., Peltier, W.R., Tan, N., Contoux, C., Ramstein, G., Li, X., Zhang, Z., Guo, C., Nisancioglu, K.H., Zhang, Q., Li, Q., Kamae, Y., Chandler, M.A., Sohl, L.E., Otto-Bliesner, B.L., Feng, R., Brady, E.C., von der Heydt, A.S., Baatsen, M.L.J., Lunt, D.J., 2020. The Pliocene Model Intercomparison Project Phase 2: large-scale climate features and climate sensitivity. Climate of the Past 16, 2095-2123.

Hearty, P., Hollin, J., Neumann, A., O'Leary, M., McCulloch, M., 2007. Global sea-level fluctuations during the Last Interglaciation (MIS 5e). Quaternary Science Reviews 26, 2090-2112.

Hearty, P.J., Rovere, A., Sandstrom, M.R., O'Leary, M.J., Roberts, D., Raymo, M.E., 2020. Pliocene-Pleistocene Stratigraphy and Sea-Level Estimates, Republic of South Africa With Implications for a 400 ppmv CO2 World. Paleoceanography and Paleoclimatology 35.

Hoffman, J.S., Clark, P.U., Parnell, A.C., He, F., 2017. Regional and global sea-surface temperatures during the last interglaciation. Science 355, 276-279.

Jouzel, J., MassonDelmotte, V., Cattani, O., Dreyfus, G., Falourd, S., Hoffmann, G., Minster, B., Nouet, J., Barnola, J.M., Chappellaz, J., Fischer, H., Gallet, J.C., Johnsen, S., Leuenberger, M., Loulergue, L., Luethi, D., Oerter, H., Parrenin, F., Raisbeck, G., Raynaud, D., Schilt, A., Schwander, J., Selmo, E., Souchez, R., Spahni, R., Stauffer, B., Steffensen, J.P., Stenni, B., Stocker, T.F., Tison, J.L., Werner, M., Wolff, E.W., 2007. Orbital and Millennial Antarctic Climate Variability over the Past 800,000 Years. Science 317, 793-796.

Koenig, S.J., Dolan, A.M., de Boer, B., Stone, E.J., Hill, D.J., DeConto, R.M., Abe-Ouchi, A., Lunt, D.J., Pollard, D., Quiquet, A., Saito, F., Savage, J., van de Wal, R., 2015. Ice sheet model dependency of the simulated Greenland Ice Sheet in the mid-Pliocene. Climate of the Past 11, 369-381.

Kopp, R., Simons, F., Mitrovica, J., Maloof, A., 2009. Probabilistic assessment of sea level during the last interglacial stage. Nature.

Kopp, R.E., Simons, F.J., Mitrovica, J.X., Maloof, A.C., Oppenheimer, M., 2013. A probabilistic assessment of sea level variations within the last interglacial stage. Geophysical Journal International 193, 711-716.

Lang, N., Wolff, E.W., 2011. Interglacial and glacial variability from the last 800 ka in marine, ice and terrestrial archives. Climate of the Past 7, 361-380.

Lau, S.C.Y., Wilson, N.G., Golledge, N.R., Naish, T.R., Watts, P.C., Silva, C.N.S., Cooke, I.R., Allcock, A.L., Mark, F.C., Linse, K., Strugnell, J.M., 2023. Geonomic evidence for West Antarctic Ice Sheet collapse during the Last Interglacial. Science 382, 1384-1389.

Lüthi, D., Le Floch, M., Bereiter, B., Blunier, T., Barnola, J.-M., Siegenthaler, U., Raynaud, D., Jouzel, J., Fischer, H., Kawamura, K., Stocker, T.F., 2008. High-resolution carbon dioxide concentration record 650,000–800,000 years before present. Nature 453, 379-382.

Martinez-Boti, M.A., Foster, G.L., Chalk, T.B., Rohling, E.J., Sexton, P.F., Lunt, D.J., Pancost, R.D., Badger, M.P., Schmidt, D.N., 2015. Plio-Pleistocene climate sensitivity evaluated using high-resolution CO2 records. Nature 518, 49-54.

Masson-Delmotte, V., Stenni, B., Pol, K., Braconnot, P., Cattani, O., Falourd, S., Kageyama, M., Jouzel, J., Landais, A., Minster, B., Barnola, J.M., Chappellaz, J., Krinner, G., Johnsen, S., Röthlisberger, R., Hansen, J., Mikolajewicz, U., Otto-Bliesner, B., 2010. EPICA Dome C record of glacial and interglacial intensities. Quaternary Science Reviews 29, 113-128.

McKay, N., Overpeck, J., 2011. The role of ocean thermal expansion in Last Interglacial sea level rise. Geophysical Research ….

Melles, M., Brigham-Grette, J., Minyuk, P.S., Nowaczyk, N.R., Wennrich, V., DeConto, R.M., Anderson, P.M., Andreev, A.A., Coletti, A., Cook, T.L., Haltia-Hovi, E., Kukkonen, M., Lozhkin, A.V., Rosen, P., Tarasov, P., Vogel, H., Wagner, B., 2012. 2.8 Million Years of Arctic Climate Change from Lake El'gygytgyn, NE Russia. Science 337, 315-320.

Miller, K.G., Wright, J.D., Browning, J.V., Kulpecz, A., Kominz, M., Naish, T.R., Cramer, B.S., Rosenthal, Y., Peltier, W.R., Sosdian, S., 2012. High tide of the warm Pliocene: Implications of global sea level for Antarctic deglaciation. Geology 40, 407-410.

Morlighem, M., Williams, C.N., Rignot, E., An, L., Arndt, J.E., Bamber, J.L., Catania, G., Chauche, N., Dowdeswell, J.A., Dorschel, B., Fenty, I., Hogan, K., Howat, I., Hubbard, A., Jakobsson, M., Jordan, T.M., Kjeldsen, K.K., Millan, R., Mayer, L., Mouginot, J., Noel, B.P.Y., O'Cofaigh, C., Palmer, S., Rysgaard, S., Seroussi, H., Siegert, M.J., Slabon, P., Straneo, F., van den Broeke, M.R., Weinrebe, W., Wood, M., Zinglersen, K.B., 2017. BedMachine v3: Complete Bed Topography and Ocean Bathymetry Mapping of Greenland From Multibeam Echo Sounding Combined With Mass Conservation. Geophys Res Lett 44, 11051-11061.

Moucha, R., Ruetenik, G.A., 2017. Interplay between dynamic topography and flexure along the U.S. Atlantic passive margin: Insights from landscape evolution modeling. Global and Planetary Change 149, 72-78.

Muhs, D., Simmons, K., Steinke, B., 2002a. Timing and warmth of the Last Interglacial period: new U-series evidence from Hawaii and Bermuda and a new fossil compilation for North America. Quaternary Science Reviews 21, 1355-1383.

Muhs, D., 2002b. Evidence for the Timing and Duration of the Last Interglacial Period from High-Precision Uranium-Series Ages of Corals on Tectonically Stable Coastlines. Quaternary Research 58, 36-40.

Muhs, D., Simmons, K., Schumann, R., 2011. Sea-level history of the past two interglacial periods: new evidence from U-series dating of reef corals from south Florida. Quaternary Science Reviews 30, 570-590.

NEEM Community Members, Dahl-Jensen, D., Albert, M.R., Aldahan, A., Azuma, N., Balslev-Clausen, D., Baumgartner, M., Berggren, A.M., Bigler, M., Binder, T., Blunier, T., Bourgeois, J.C., Brook, E.J., Buchardt, S.L., Buizert, C., Capron, E., Chappellaz, J., Chung, J., Clausen, H.B., Cvijanovic, I., Davies, S.M., Ditlevsen, P., Eicher, O., Fischer, H., Fisher, D.A., Fleet, L.G., Gfeller, G., Gkinis, V., Gogineni, S., Goto-Azuma, K., Grinsted, A., Gudlaugsdottir, H., Guillevic, M., Hansen, S.B., Hansson, M., Hirabayashi, M., Hong, S., Hur, S.D., Huybrechts, P., Hvidberg, C.S., Iizuka, Y., Jenk, T., Johnsen, S.J., Jones, T.R., Jouzel, J., Karlsson, N.B., Kawamura, K., Keegan, K., Kettner, E., Kipfstuhl, S., Kjær, H.A., Koutnik, M., Kuramoto, T., Köhler, P., Laepple, T., Landais, A., Langen, P.L., Larsen, L.B., Leuenberger, D., Leuenberger, M., Leuschen, C., Li, J., Lipenkov, V., Martinerie, P., Maselli, O.J., MassonDelmotte, V., McConnell, J.R., Miller, H., Mini, O., Miyamoto, A., Montagnat-Rentier, M., Mulvaney, R., Muscheler, R., Orsi, A.J., Paden, J., Panton, C., Pattyn, F., Petit, J.R., Pol, K., Popp, T., Possnert, G., Prié, F., Prokopiou, M., Quiquet, A., Rasmussen, S.O., Raynaud, D., Ren, J., Reutenauer, C., Ritz, C., Röckmann, T., Rosen, J.L., Rubino, M., Rybak, O., Samyn, D., Sapart, C.J., Schilt, A., Schmidt, A.M.Z., Schwander, J., Schüpbach, S., Seierstad, I., Severinghaus, J.P., Sheldon, S., Simonsen, S.B., Sjolte, J., Solgaard, A.M., Sowers, T., Sperlich, P., Steen-Larsen, H.C., Steffen, K., Steffensen, J.P., Steinhage, D., Stocker, T.F., Stowasser, C., Sturevik, A.S., Sturges, W.T., Sveinbjörnsdottir, A., Svensson, A., Tison, J.L., Uetake, J., Vallelonga, P., Van De Wal, R.S.W., van der Wel, G., Vaughn, B.H., Vinther, B., Waddington, E., Wegner, A., Weikusat, I., White, J.W.C., Wilhelms, F., Winstrup, M., Witrant, E., Wolff, E.W., Xiao, C., Zheng, J., 2013. Eemian interglacial reconstructed from a Greenland folded ice core. Nature 493, 489-494.O'Leary, M.J., Hearty, P.J., Thompson, W.G., Raymo, M.E., Mitrovica, J.X., Webster, J.M., 2013.

Ice sheet collapse following a prolonged period of stable sea level during the last interglacial. Nature Geoscience 6, 796-800.

Otto-Bliesner, B.L., Rosenbloom, N., Stone, E.J., McKay, N.P., Lunt, D.J., Brady, E.C., Overpeck, J.T., 2013. How warm was the last interglacial? New model-data comparisons. Philosophical Transactions of the Royal Society A: Mathematical, Physical and Engineering Sciences 371, 20130097-20130097.

Pagani, M., Liu, Z., LaRiviere, J., Ravelo, A.C., 2010. High Earth-system climate sensitivity determined from Pliocene carbon dioxide concentrations. Nature Geoscience 3, 27-30.

Polyak, V.J., Onac, B.P., Fornós, J.J., Hay, C., Asmerom, Y., Dorale, J.A., Ginés, J., Tuccimei, P., Ginés, A., 2018. A highly resolved record of relative sea level in the western Mediterranean Sea during the last interglacial period. Nature Geoscience 11, 860-864.

Raymo, M., Kozdon, R., Evans, D., Lisiecki, L., Ford, H.L., 2018. The accuracy of mid-Pliocene δ18O-based ice volume and sea level reconstructions. Earth-Science Reviews 177, 291-302.

Raymo, M.E., Mitrovica, J.X., 2012. Collapse of polar ice sheets during the stage 11 interglacial. Nature 483, 453-456.

Reyes, A.V., Carlson, A.E., Beard, B.L., Hatfield, R.G., Stoner, J.S., Winsor, K., Welke, B., Ullman, D.J., 2014. South Greenland ice-sheet collapse during Marine Isotope Stage 11. Nature 510, 525-528.

Richards, F.D., Coulson, S.L., Hoggard, M.J., Austermann, J., Dyer, B., Mitrovica, J.X., 2023. Geodynamically corrected Pliocene shoreline elevations in Australia consistent with midrange projections of Antarctic ice loss. Science Advances 9, eadg3035.

Roberts, D.L., Karkanas, P., Jacobs, Z., Marean, C.W., Roberts, R.G., 2012. Melting ice sheets 400,000 yr ago raised sea level by 13m: Past analogue for future trends. Earth and Planetary Science Letters 357-358, 226-237.

Robinson, A., Alvarez-Solas, J., Calov, R., Ganopolski, A., Montoya, M., 2017. MIS-11 duration key to disappearance of the Greenland ice sheet. Nat Commun 8, 16008.

Rohling, E.J., Hibbert, F.D., Grant, K.M., Galaasen, E.V., Irvali, N., Kleiven, H.F., Marino, G., Ninnemann, U., Roberts, A.P., Rosenthal, Y., Schulz, H., Williams, F.H., Yu, J., 2019. Asynchronous Antarctic and Greenland ice-volume contributions to the last interglacial sea-level highstand. Nat Commun 10, 5040.

Rovere, A., Raymo, M.E., Mitrovica, J.X., Hearty, P.J., O'Leary, M.J., Inglis, J.D., 2014. The Mid-Pliocene sea-level conundrum: Glacial isostasy, eustasy and dynamic topography. Earth and Planetary Science Letters 387, 27-33.

Seki, O., Foster, G.L., Schmidt, D.N., Mackensen, A., Kawamura, K., 2010. Alkenone and boron-based Pliocene pCO2 records. Earth and Planetary Science Letters 292, 201-211.

Shackleton, S., Baggenstos, D., Menking, J.A., Dyonisius, M.N., Bereiter, B., Bauska, T.K., Rhodes, R.H., Brook, E.J., Petrenko, V.V., McConnell, J.R., Kellerhals, T., Häberli, M., Schmitt, J., Fischer, H., Severinghaus, J.P., 2020. Global ocean heat content in the Last Interglacial. Nature Geoscience 13, 77-81.

Stirling, C., Esat, T., Lambeck, K., 1998. Timing and duration of the Last Interglacial: evidence for a restricted interval of widespread coral reef growth. Earth and Planetary ….

Thompson, W., Curran, H., Wilson, M., 2011. Sea-level oscillations during the last interglacial highstand recorded by Bahamas corals. Nature Geoscience 4, 684-687.

Turney, C.S.M., Fogwill, C.J., Golledge, N.R., McKay, N.P., van Sebille, E., Jones, R.T., Etheridge, D., Rubino, M., Thornton, D.P., Davies, S.M., Ramsey, C.B., Thomas, Z.A., Bird, M.I., Munksgaard, N.C., Kohno, M., Woodward, J., Winter, K., Weyrich,

L.S., Rootes, C.M., Millman, H., Albert, P.G., Rivera, A., van Ommen, T., Curran, M., Moy, A., Rahmstorf, S., Kawamura, K., Hillenbrand, C.D., Weber, M.E., Manning, C.J., Young, J., Cooper, A., 2020. Early Last Interglacial ocean warming drove substantial ice mass loss from Antarctica. Proc Natl Acad Sci U S A 117, 3996-4006.

Veeh, H.H., 1966. Th230/U238 and U234/U238 ages of Pleistocene high sea level stand. Journal of Geophysical Research 71, 3379-3386.

Vyverberg, K., Dechnik, B., Dutton, A., Webster, J.M., Zwartz, D., Portell, R.W., 2018. Episodic reef growth in the granitic Seychelles during the Last Interglacial: Implications for polar ice sheet dynamics. Marine Geology 399, 170-187.

Wardlaw, B.R., Quinn, T.M., 1991. The record of Pliocene sea-level change at Enewetak Atoll. Quaternary Science Reviews 10, 247-258.

Yau, A.M., Bender, M.L., Robinson, A., Brook, E.J., 2016. Reconstructing the last interglacial at Summit, Greenland: Insights from GISP2. Proc Natl Acad Sci U S A 113, 9710-9715.
